# Based on molecular docking and real-time PCR technology, the two-component system Bae SR was investigated on the mechanism of drug resistance in CRAB

**DOI:** 10.1186/s12866-024-03286-5

**Published:** 2024-04-15

**Authors:** Beizhen Pan, Yuefeng Wang, Jiansheng Su, Yan Liu, Jifei Yang, Yujiao Zhou, Liyuan Sun

**Affiliations:** School of Medical Technology, Beihua University, No. 3999 Binjiang East Road, Fengman District, Jilin, Jilin Province China

**Keywords:** *Acinetobacter baumannii*, Carbapenemase, Molecular docking, Two-component system

## Abstract

This study aimed to explore the role of the two-component system Bae SR in the mechanism of drug resistance in carbapenem-resistant *A. baumannii* (CRAB) using molecular docking and real-time polymerase chain reaction (PCR). The two-component system Bae SR of *Acinetobacter baumannii* was subjected to molecular docking with imipenem, meropenem, and levofloxacin. Antibacterial assays and fluorescence quantitative PCR were used to explore protein–ligand interactions and molecular biological resistance mechanisms related to CRAB. The analysis of the two-component system in *A. baumannii* revealed that imipenem exhibited the highest docking energy in Bae S at − 5.81 kcal/mol, while the docking energy for meropenem was − 4.92 kcal/mol. For Bae R, imipenem had a maximum docking energy of − 4.28 kcal/mol, compared with − 4.60 kcal/mol for meropenem. The highest binding energies for Bae S–levofloxacin and Bae R–levofloxacin were − 3.60 and − 3.65 kcal/mol, respectively. All imipenem-resistant strains had minimum inhibitory concentration (MIC) values of 16 µg/mL, whereas levofloxacin-resistant strains had MIC values of 8 µg/mL. The time-sterilization curve showed a significant decrease in bacterial colony numbers at 2 h under the action of 8 µg/mL imipenem, indicating antibacterial effects. In contrast, levofloxacin did not exhibit any antibacterial activity. Fluorescence quantitative PCR results revealed significantly increased relative expression levels of *bae S* and *bae R* genes in the CRAB group, which were 2 and 1.5 times higher than those in the CSAB group, respectively, with statistically significant differences. Molecular docking in this study found that the combination of Bae SR and carbapenem antibiotics (imipenem, meropenem) exhibited stronger affinity and stability compared with levofloxacin. Moreover, the overexpression of the two-component system genes in carbapenem-resistant *A. baumannii* enhanced its resistance to carbapenem, providing theoretical and practical insights into carbapenem resistance in respiratory tract infections caused by *A. baumannii*.

## Introduction

On a global scale, “*ESKAPE*” resistance has become a significant contributor to disease and mortality in patients [1]. Among these, *Acinetobacter baumannii* is the most common non-fermenting gram-negative bacterium, often responsible for hospital-acquired infections, particularly respiratory tract infections. Empirical antibiotic treatment has led to the emergence of multiple resistance and widespread resistance [2]. Carbapenems serve as the last line of defense for treating infections, but the resistance rate of *A. baumannii* to carbapenems has shown a significant upward trend due to their increased clinical usage and intensity [3].

Studies on the current resistance mechanisms of carbapenem-resistant *A. baumannii* (CRAB) primarily focus on both “enzymatic” and “non-enzymatic” aspects. Enzymes produce carbapenemases, categorized into three classes: A, B, and D, which are primarily detected by phenotype and genotype methods. The “non-enzymatic” drug resistance mechanisms involve “target sites” and “membranes”, corresponding to different antibiotics and exhibiting various mutation characteristics. The term “membrane” includes phenomena such as overexpression of efflux pumps, decreased or absent expression of outer membrane proteins, and biofilm formation [4].

Two-component systems (TCSs) represent crucial signal transduction systems in bacteria, playing significant roles in antibiotic resistance and virulence regulation. They can confer drug resistance by modifying cell surfaces, reducing drug infiltration or increasing drug efflux, and promoting biofilm formation [5, 6]. The two-component system Bae RS, which was first discovered by Raffa and Raivio in *Escherichia coli*, acts as an extracellular stress response system regulating the *Mdt ABCD* efflux pump [7]. Studies have found that the biological functions of *Ade ABC* and *Mdt ABCD* in *A. baumannii* resemble those in *E. coli*. Therefore, Bae RS can regulate the expression of the *Ade ABC* gene in the efflux pump of *A. baumannii*, thereby enhancing drug resistance [8]. The gradual development of molecular docking technology in recent years has led to the emergence of a new approach for studying the interaction between protein molecules, offering insights into bacterial drug resistance mechanisms [9–11]. Therefore, it is of great significance to explore the molecular mechanism of CRAB resistance in a two-component system, providing a theoretical and practical basis for carbapenem resistance in respiratory infections caused by *A. baumannii* in clinical practice, and offering new ideas for the clinical treatment of *A. baumannii* infections.

## Materials and methods

### Specimen source

This study used 35 strains of *A. baumannii*, comprising 10 strains categorized as CSAB and 25 strains classified as CRAB, including those with levofloxacin resistance.

### Instruments and reagents

The main instruments used in the study included a mold incubator (MJX-160B-Z; Shanghai Boxun Industrial Co., Ltd.), a biological safety cabinet (BSC-1600II.B2; Shanghai Sujing Industrial Co., Ltd.), a bacterial turbidity meter (WGZ-2XJ; Shanghai Xinrui Instrument Co., Ltd.), and a Piko Real real-time PCR instrument (TCR0024; Thermo Fisher Scientific, USA).

The main reagents used were 2×RealStar Power SYBR qPCR Mix (GeneStar) and imipenem and levofloxacin (Shanghai Yuanye Biotechnology Co., Ltd.).

### Server and tools

This study conducted all bioinformatics analyses using SWISS-MODEL (https://swissmodel.expasy.org/) and the PubChem database (http://pubchem.ncbi.nlm.nih.gov/). Molecular docking was performed using Autodock, and visualization of docking complexes was performed using Pymol.

### Preparation of receptor proteins

Receptor proteins were prepared by querying the protein sequences of Bae S and Bae R from the National Center for Biotechnology Information (NCBI) database. The entry sequences were WP_012300504.1 (https://www.ncbi.nlm.nih.gov/protein/WP_012300504.1) and WP_000680574.1 (https://www.ncbi.nlm.nih.gov/protein/WP_000680574.1), respectively. The secondary structure was predicted using the PSIPRED database (http://bioinf.cs.ucl.ac.uk/psipred/), whereas the NetPhos-3.1 server (https://services.healthtech.dtu.dk/) was used to predict serine, threonine, and tyrosine sites. The SWISS-MODEL was used to build the three-dimensional (3D) structure models of Bae S and Bae R. Template sequences numbered 7CCH (https://www.rcsb.org/structure/7CCH) 4B09 (https://www.rcsb.org/structure/4B09) were forecasted, and PDB files were downloaded. Autodock software was used for hydrogenation, charge calculation, and atomic type setting of receptor proteins. In addition, the VADAR 1.8 online server (http://vadar.wishartlab.com/) was used to calculate the statistical percentage of protein secondary structure, generate the Ramachandran graph [12], evaluate the percentage values of the optional and preferred regions in the graph.

### Preparation of small-molecule ligands

The 3D structures of imipenem, meropenem, and levofloxacin were retrieved from the PubChem database using their respective PubChem IDs: 104,838 (https://pubchem.ncbi.nlm.nih.gov/compound/104838), 441,130 (https://pubchem.ncbi.nlm.nih.gov/compound/441130), and 149,096 (https://pubchem.ncbi.nlm.nih.gov/compound/149096). The structures were downloaded in SDF file format. Autodock software was used to process small-molecule ligand charge adjustments.

### Generation of receptor proteins grid and molecular docking

Specific grid cube box sizes were entered with fixed values for the *x*, *y*, and *z* axes to predict the phosphorylation sites as docking pockets. For Bae S, the grid box was fixed at *X* = 19.374, *Y* = 67.897, and *Z* = 4.26, whereas for Bae R, the grid box values were fixed at *X* = − 21.361, *Y* = − 67.564, and *Z* = − 80.515. Autodock software was used to dock the receptor protein and small-molecule ligand. Different molecular docking complexes were analyzed based on their binding energy values (kcal/moL) and hydrogen bonds. Visual results were further analyzed using Pymol.

### Determination of MIC values and generation of time–sterilization curve

A single colony was inoculated into LB liquid medium and incubated at 200 rpm and 37℃ to the logarithmic growth phase. For each well of a sterile 96-well plate,100 µL of LB liquid medium and 10 µL of overnight cultured bacterial liquid were added. Then, 100 µL of imipenem solution was added for gradient dilution to achieve final concentrations of 128, 64, 32, 16, 8, 4, 2, 1 µg/mL. The plate was then cultured at 37℃ for 18–24 h, and the bacterial growth was observed by the naked eye to determine the MIC as the lowest concentration inhibiting visible growth. In parallel, antibiotics at a final concentration of ½ MIC were added to the bacterial solution. The mixture was then incubated at 200 rpm and 37 °C on a shaker. Samples were collected at 0, 2, 4, 6, 12, and 24 h post-administration. Then, 100 µL of bacterial solution extracted each time was diluted 10 times with 0.9% normal saline 4 times, resulting in a final dilution of 1:10,000. Then, 200 µL of the diluted bacterial solution was spread onto an LB solid medium and mixed thoroughly. The plates were inverted and incubated at 37℃ for 18–24 h, and the colonies were counted. A time–sterilization curve was plotted using the administration time as the horizontal coordinate (*x*-axis) and the logarithm of the colony-forming units as the vertical coordinate (*y*-axis).

### Primer design and reverse transcription PCR detection of relative expression levels of two-component system genes *bae S* and *bae R*

RNA was extracted using the traditional Trizol method, followed by reverse transcription to obtain cDNA. The *16S rRNA* gene was used as the internal reference gene. The Ct values of the two-component system genes *bae S* and *bae R* were determined using fluorescence quantitative PCR. The reaction conditions included an initial denaturation at 95℃ for 10 min, followed by denaturation at 95 °C for 15 s, annealing at 60 °C for 1 min, and 40 cycles of amplification(refer to the instructions for GeneStar(https://www.gene-star.com/) A311 product). The formula used for calculating relative expression was as follows: ΔCt = Ct_target gene_ – Ct_internal reference gene_; ΔΔCt = ΔCt_experimental group_ – ΔCt_control group_; and gene expression = 2^–ΔΔCt^. The experiment was conducted in triplicate.

### Nucleotide sequence registration number

The nucleotide sequences of the required genes in this study were stored in the NCBI database under the GenBank accession numbers: *16S rRNA* (LN611374.1), *bae S* (MK344183.1), and *bae R* (MK344168.1). The sequence of primers used in this study is shown in Table 1.

**Table 1:** Primer sequences used in this study

Primer	Primer sequence (5’→3’)	Length (bp)
*16S rRNA*	F: CTTGCCAGCATTTCGGATGG R: TCGCTGTGTAGCAACCCTTT	146
*bae S*	F: TTCCCGCTAAGTGCAAACCA R: AGCCGGTCTGGTAAAGCAAT	122
*bae R*	F: AACGCCAACAGAATTTCGCC R: TACCAGTTTCGGCCACTTCC	176

### Statistical analysis

Each sample was replicated three times, and statistical analysis was performed using SPSS 22.0 software. A bar chart was created using GraphPad Prism 9 software. Two independent-samples *t* tests were used to compare the mRNA expression levels between the CRAB and the CSAB groups. A *P* value < 0.05 indicated a statistically significant difference, a *P* value < 0.01 indicated a significant difference, and a *P* value < 0.001 indicated an extremely significant difference.

## Results

### Modeling results of structure and homology analysis of the Bae SR protein in the two-component system

**Fig. 1:**
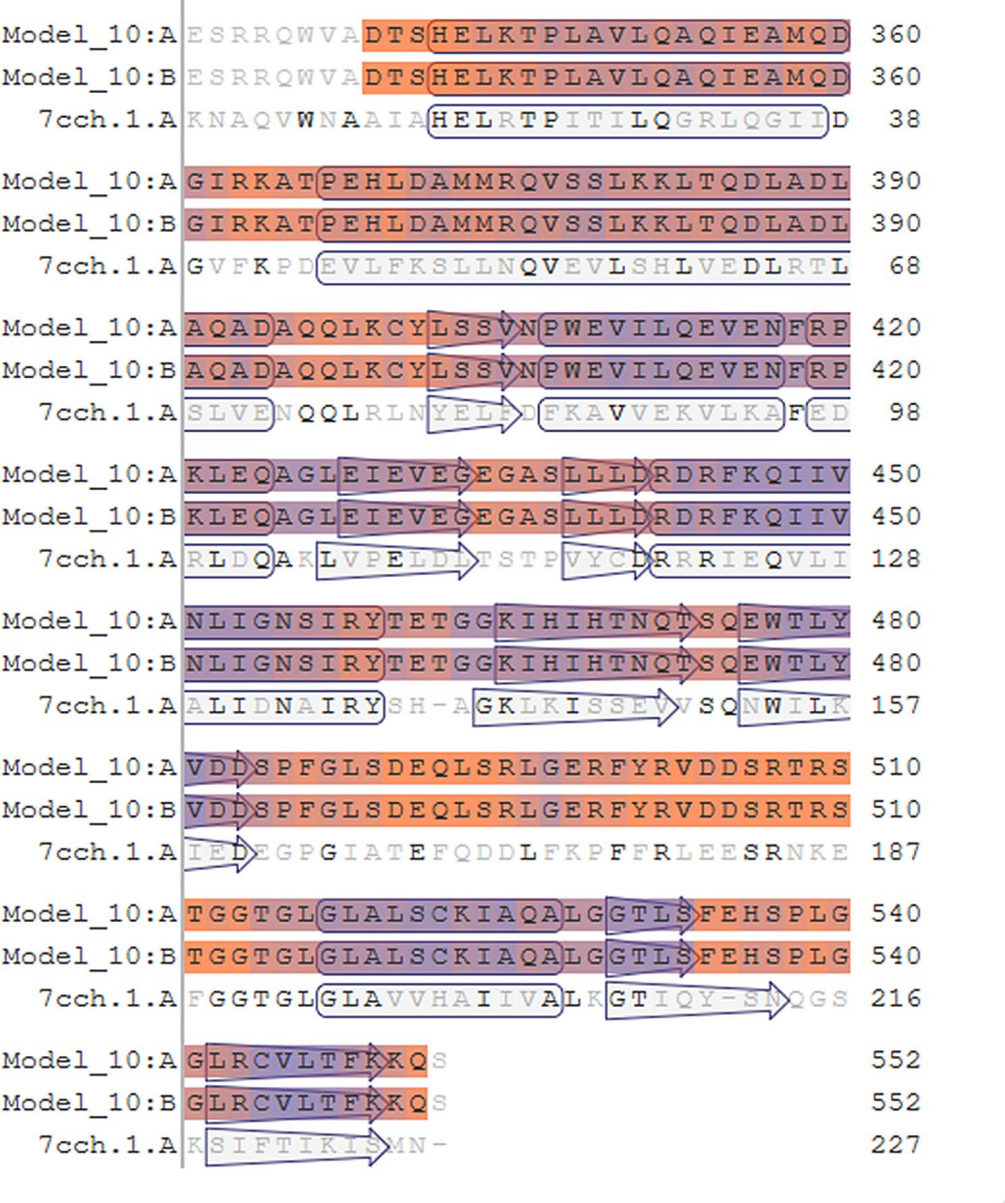
Sequence alignment between Bae S and 7CCH

**Fig. 2:**
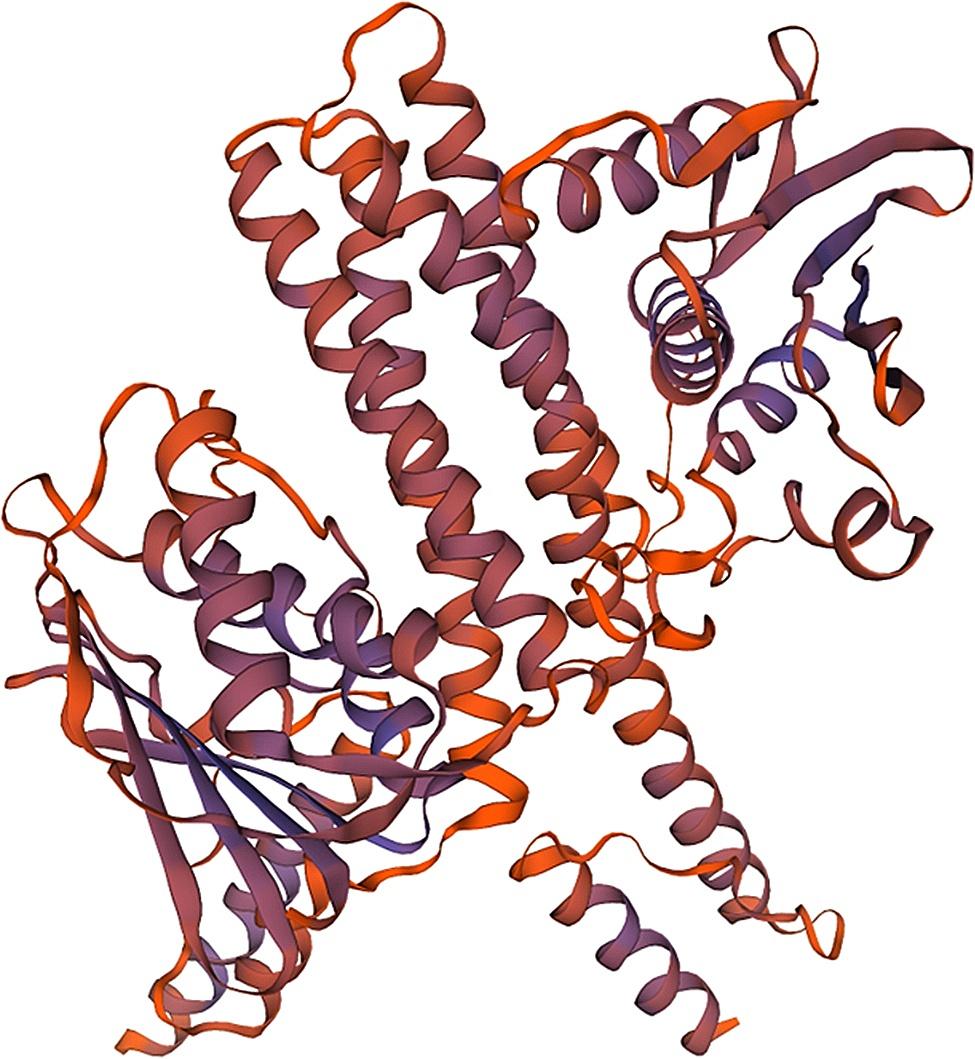
Stereochemical structure of Bae S

**Fig. 3:**
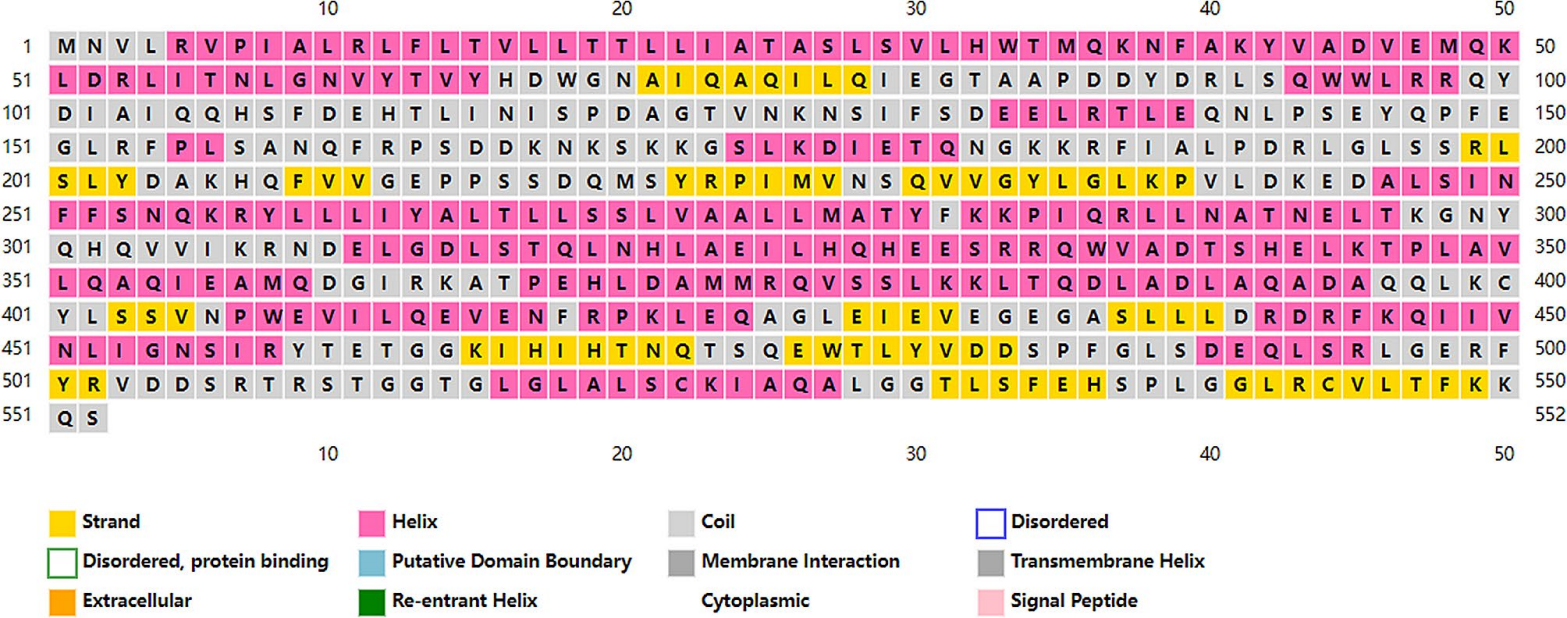
Amino acid sequence and secondary structure of Bae S. (α helix: yellow; β fold: pink; Random curl: gray)

**Fig. 4:**
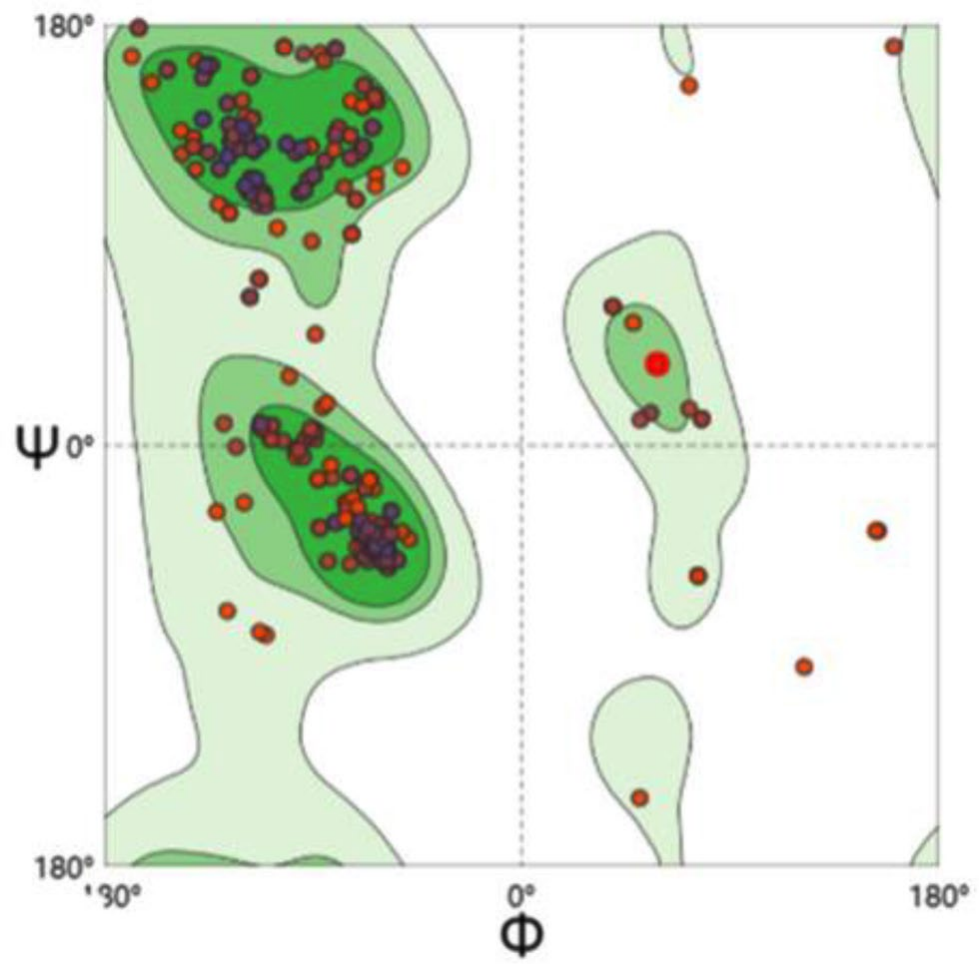
Bae S Ramachandran diagram

Bae S functions as a histidine kinase within a two-component system. A tertiary structure model was constructed using 7CCH (Fig. 1) as a template. Its 3D structure (Fig. 2) illustrates Bae S as a homodimer composed of chains A and B, totaling 552 amino acids with a molecular weight of 62.48 kDa. According to the structure analysis performed by the VADAR 1.8 online server, the Bae S protein comprised 45% α-helix, 25% β-fold, 28% coil, and 6% turns. The specific positions of amino acids corresponding to the secondary structure are shown in Fig. 3. The Ramachandran diagram of Bae S indicated that 92.65% of amino acids existed in the preferred region, 5.45% of residues existed in the allowed region, and 1.90% of residues were abnormal (Fig. 4).

**Fig. 5:**
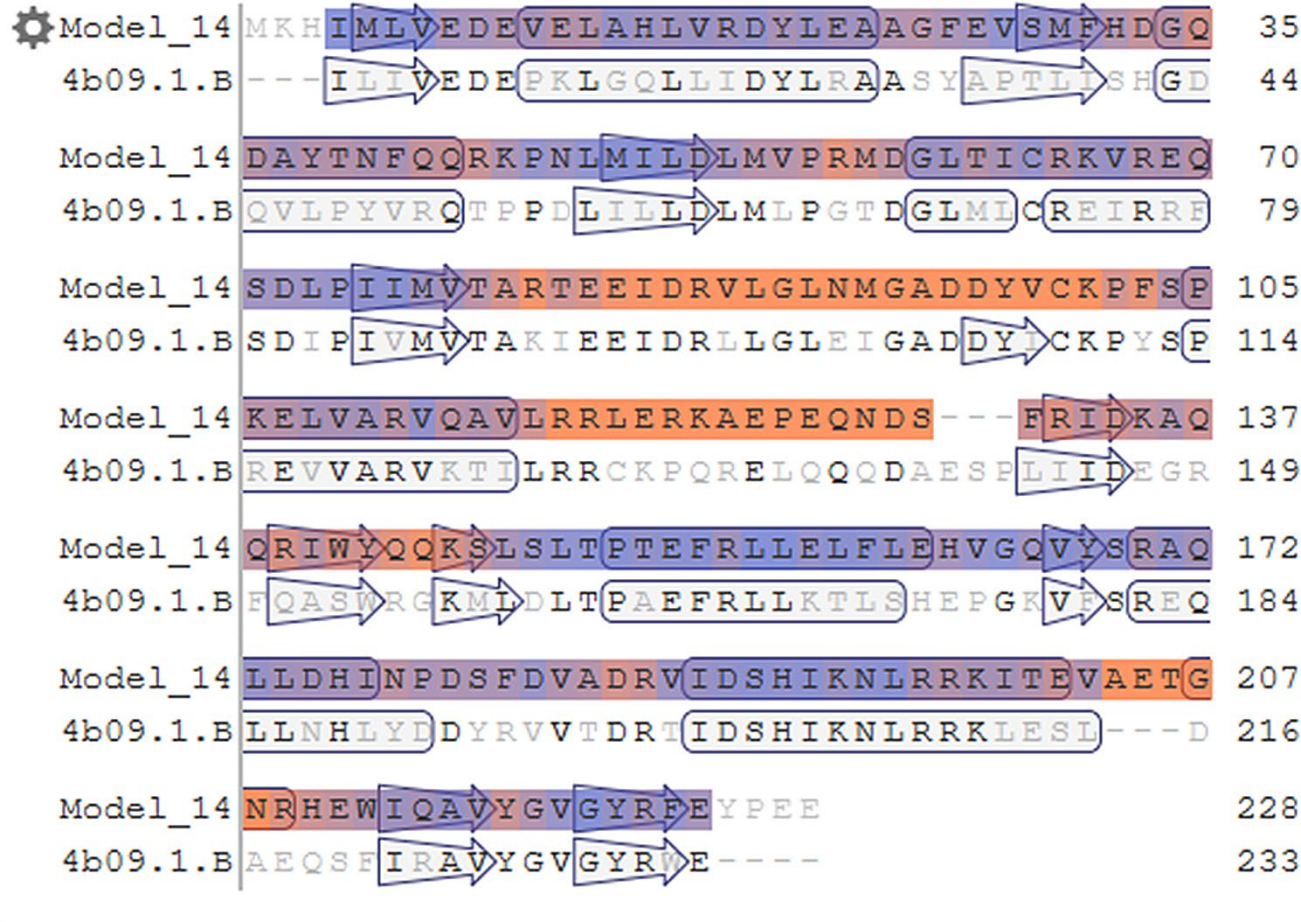
Sequence alignment between Bae R and 4B09

**Fig. 6:**
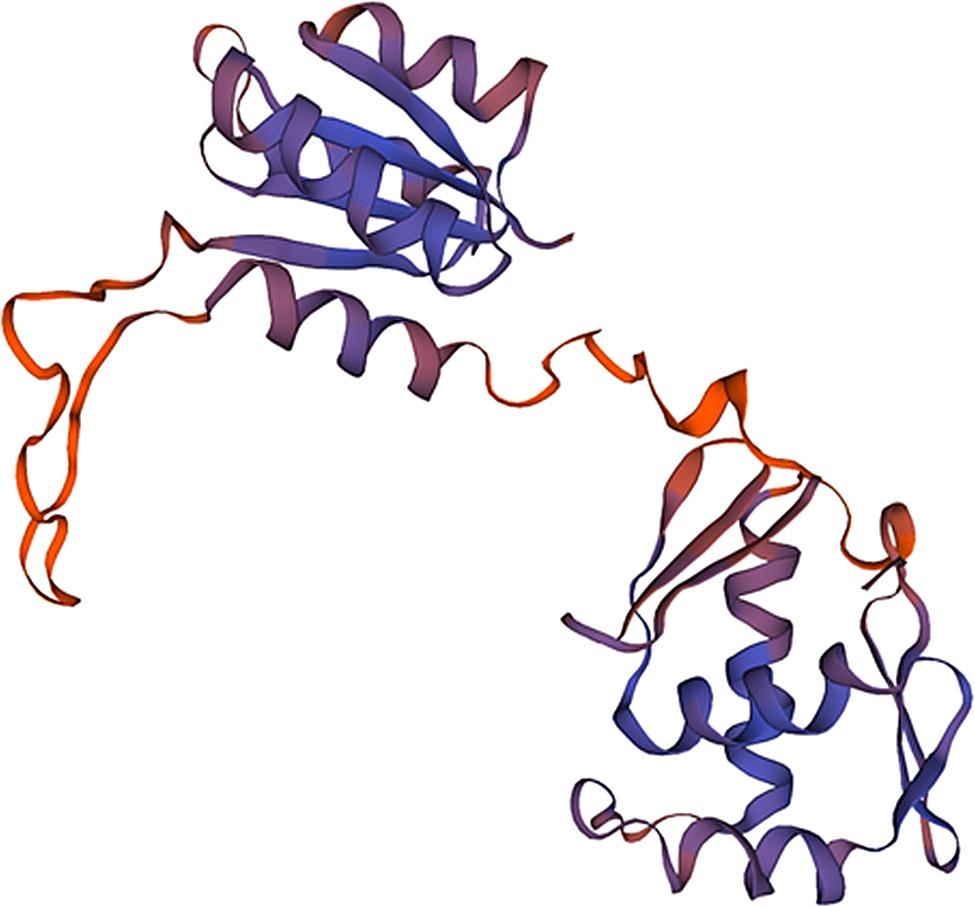
Stereochemical structure of Bae R

**Fig. 7:**
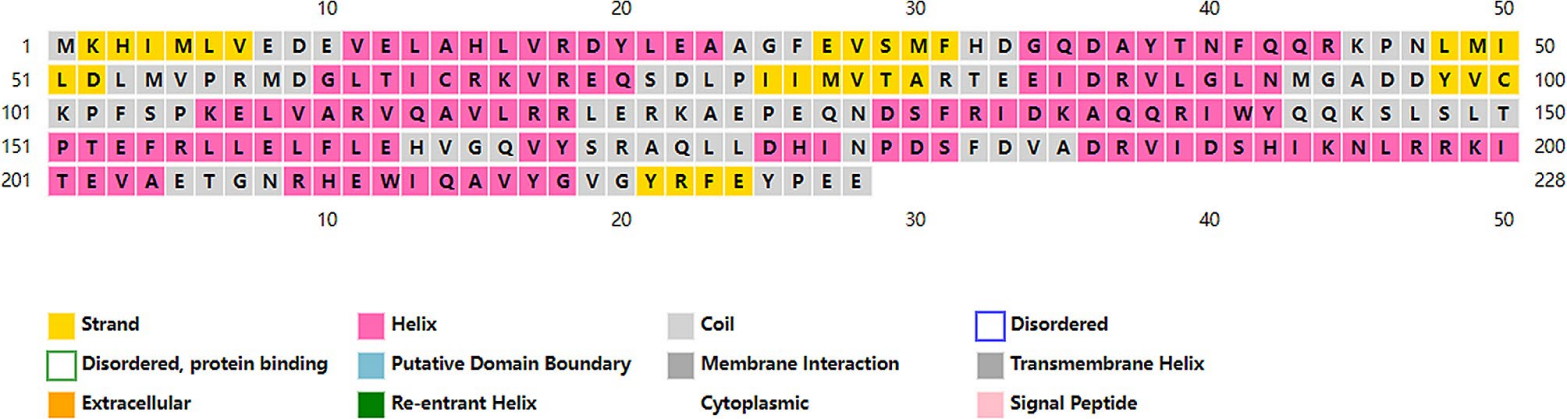
Amino acid sequence and secondary structure of Bae R. (α helix: yellow; β fold: pink; Random curl: gray)

**Fig. 8:**
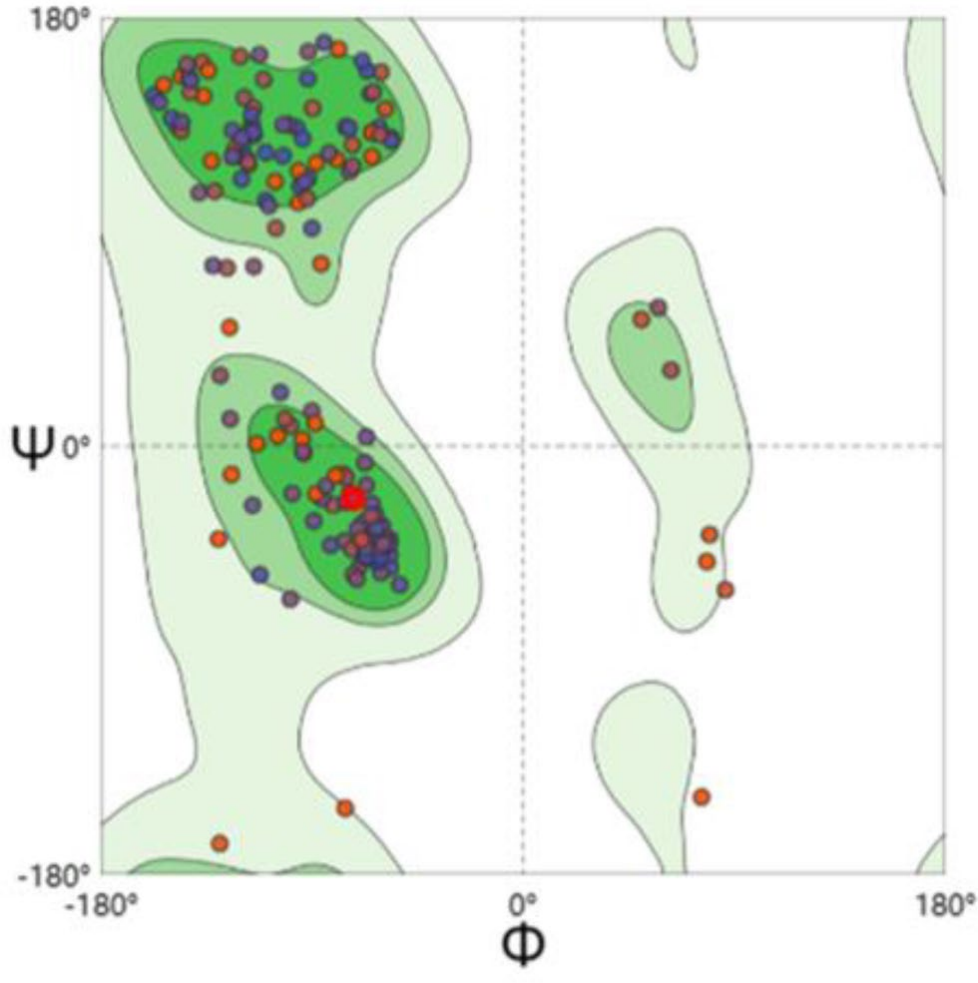
Bae R Ramachandran diagram

Bae R serves as a response regulator protein within the two-component system, with 4B09 (Fig. 5) used as a template to build its tertiary structure model. The 3D structure (Fig. 6) revealed Bae R as a monomer, consisting of a single chain of 228 amino acids with a molecular weight of 26.71 kDa. The structural analysis indicated that Bae R consisted of 34% α-helix, 24% β-strand, 40% coil, and 17% turns. The specific positions of amino acids corresponding to their secondary structure are shown in Fig. 7. The Ramachandran diagram of Bae R showed that 92.89% of amino acids existed in the preferred region, 5.33% of residues existed in the permitted region, and 1.78% of residues were abnormal (Fig. 8).

### Prediction of phosphorylation site of Bae SR in the two-component system

**Fig. 9:**
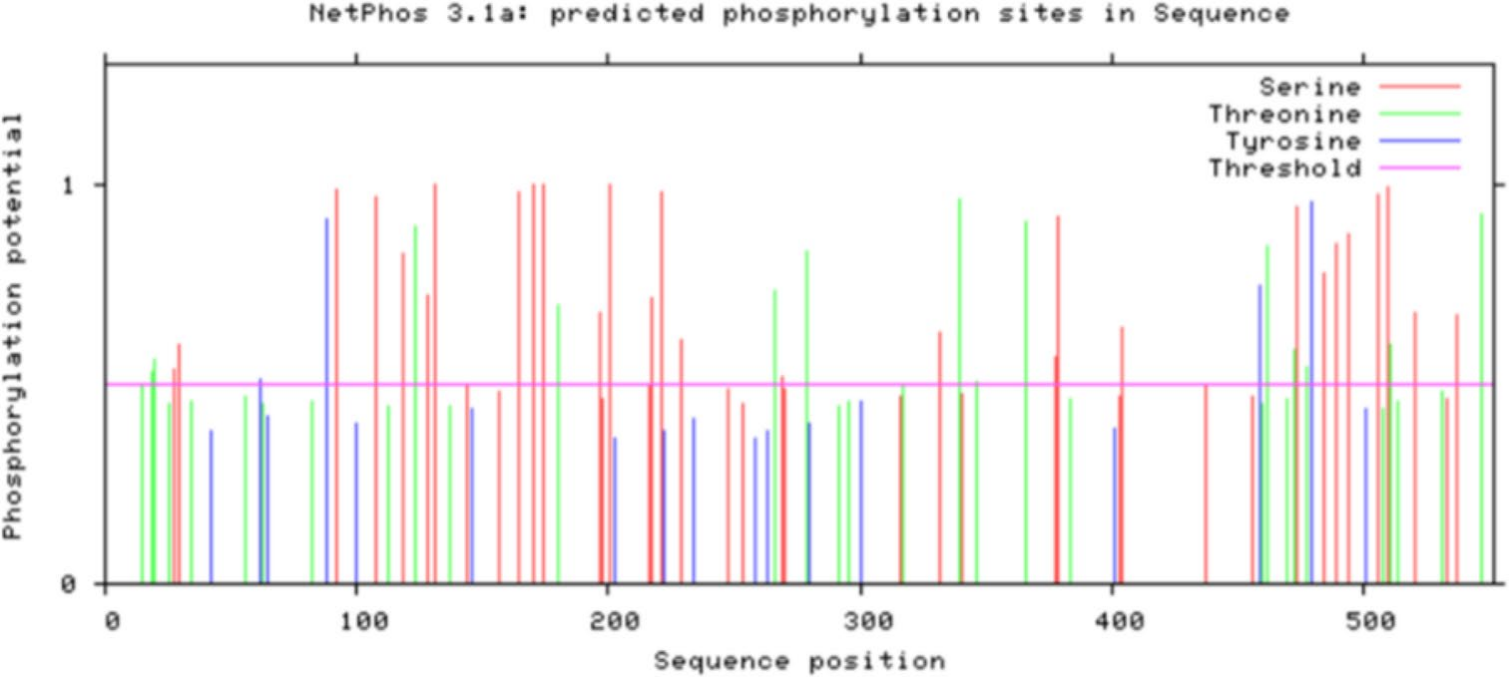
Analysis of phosphorylation sites of Bae S serine, threonine and tyrosine

**Fig. 10:**
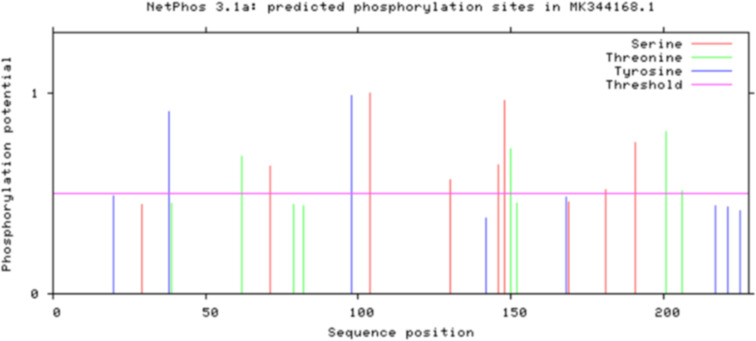
Analysis of phosphorylation sites of Bae R serine, threonine and tyrosine

The prediction of the phosphorylation site of Bae S in the HisKA domain is shown in Fig. 9. Among these, amino acids Thr 346, Tyr 459, Ser 474, and Ser 506 were identified as susceptible to autophosphorylation by enzymes in the constructed model (Table 2). Similarly, the phosphorylation site predictions for Bae R are shown in Fig. 10. Among these, amino acids Ser 71, Tyr 98, Ser 104, Thr 150, and Ser 191 were prone to autophosphorylation by enzymes in the constructed model (Table 3). The phosphorylation sites indicated in Tables 2 and 3 were used as their respective molecular docking pockets.

**Table 2:** Prediction of Bae S phosphorylation sites (score > 0.5000)

Sequence	Kinase	Score
Thr 346	P38MAPK	0.507
Tyr 459	unsp	0.747
Ser 474	unsp	0.940
Ser 506	unsp	0.976

**Table 3:** Prediction of Bae R phosphorylation sites (score > 0.6000)

Sequence	Kinase	Score
Ser 71	PKA	0.635
Tyr 98	unsp	0.983
Ser 104	unsp	0.998
Thr 150	unsp	0.723
Ser 191	PKC	0.753

### Structure of the ligand

**Fig. 11:**
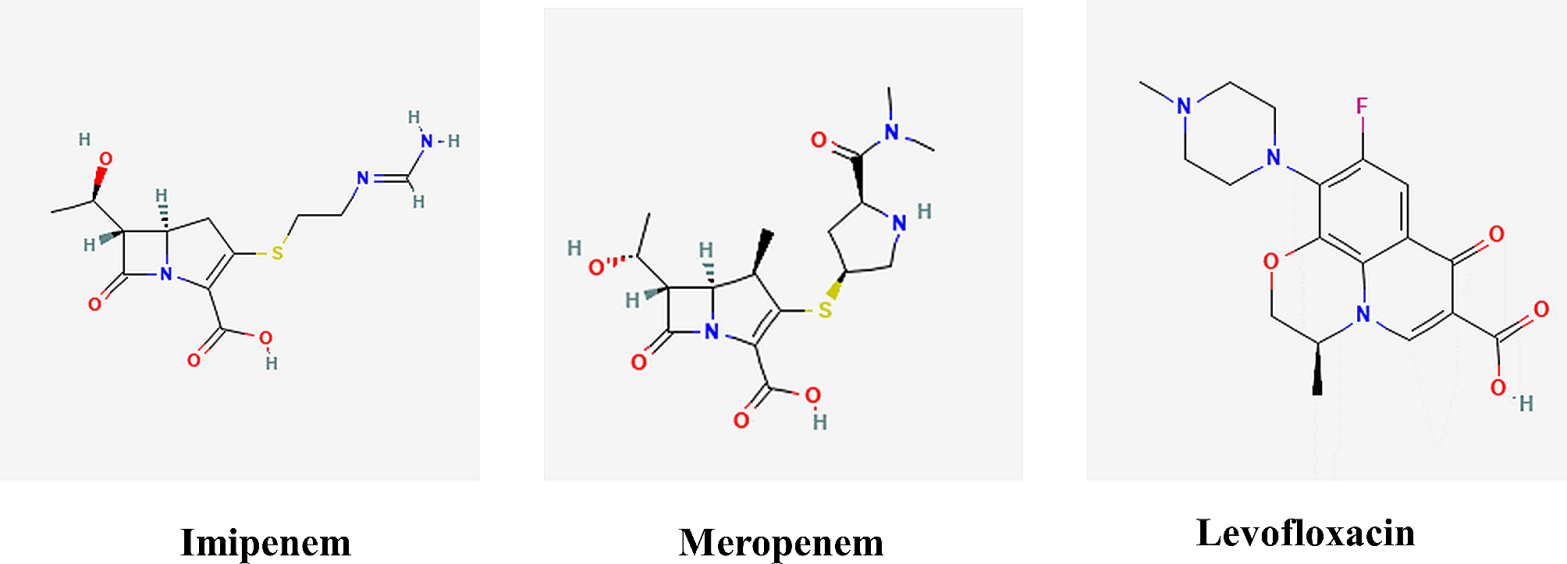
The 3D image of the ligand

The specific content of the 3D image of the ligands is shown in Fig. 11. Imipenem (C_12_H_17_N_3_O_4_S) (PubChem ID 104,838) is a widely used carbapenem antibiotic in clinical practice. It belongs to the class of antibiotics with carbapenem rings, exhibiting a broad antibacterial spectrum and potent antibacterial effects. Imipenem is mainly used for treating respiratory tract infections caused by both gram-positive and gram-negative bacteria, as well as anaerobic bacteria [13]. Meropenem (C_17_H_25_N_3_O_5_S) (PubChem ID 441,130) is a synthetic and injectable broad-spectrum carbapenem antibiotic. It functions by binding to penicillin-binding proteins on the cell wall, exerting its antibacterial effects. Meropenem is mainly used to treat severe nosocomical infections caused by gram-negative bacteria, with good stability [14]. Levofloxacin (C_18_H_20_FN_3_O_4_) (PubChem ID 149,096) mainly inhibits bacterial DNA replication and transcription, targeting DNA gyrase [15] and topoisomerase IV [16]. Levofloxacin exhibits favorable in vitro activity with minimal side effects.

### Combining docking pocket analysis with ligand interaction

**Fig. 12:**
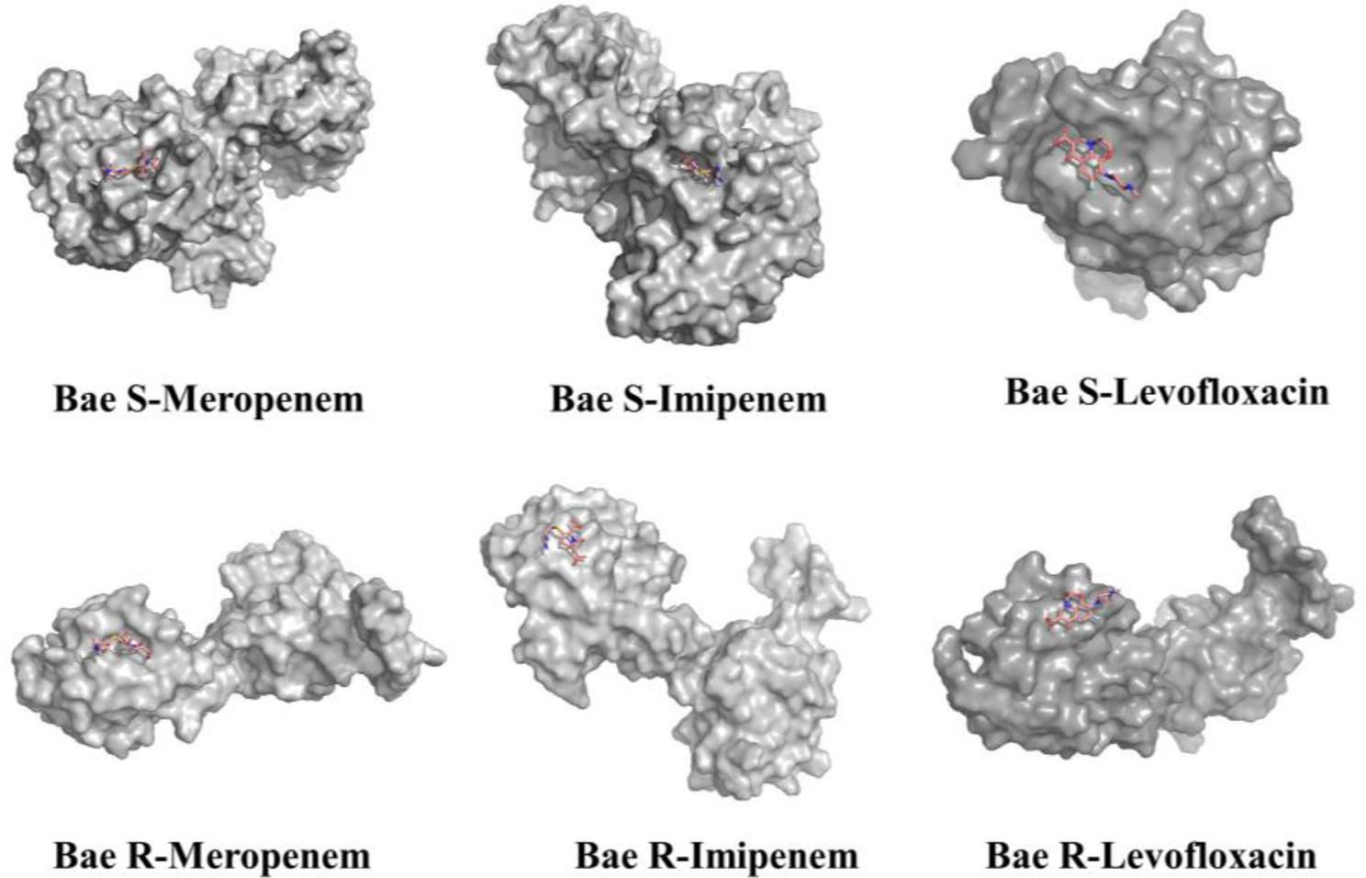
The docking results of all ligands and receptor proteins

The docking results revealed that all ligands were bound to the phosphorylation site of the receptor protein. The analysis of all docking complexes indicated that the ligands exhibited similar conformational structures, resulting in comparable binding interaction modes (Fig. 12).

Binding energy analysis: Table 4 depicts the docking results of imipenem, meropenem, and levofloxacin with the two-component system Bae SR, confirming their corresponding binding energies. The results of binding energy show that the combination of Bae SR and carbapenems (imipenem and meropenem) had a greater stability compared with levofloxacin.

**Table 4:** Docking energy of Bae R, Bae S, and carbapenems

Receptor protein	Binding energy (kcal/moL)		
	Imipenem	Meropenem	Levofloxacin
Bae S	–5.81	–4.92	–3.60
Bae R	–4.28	–4.60	–3.65

*Note* Binding energy of ≤–4 kcal/moL indicates a stable structure

**Fig. 13:**
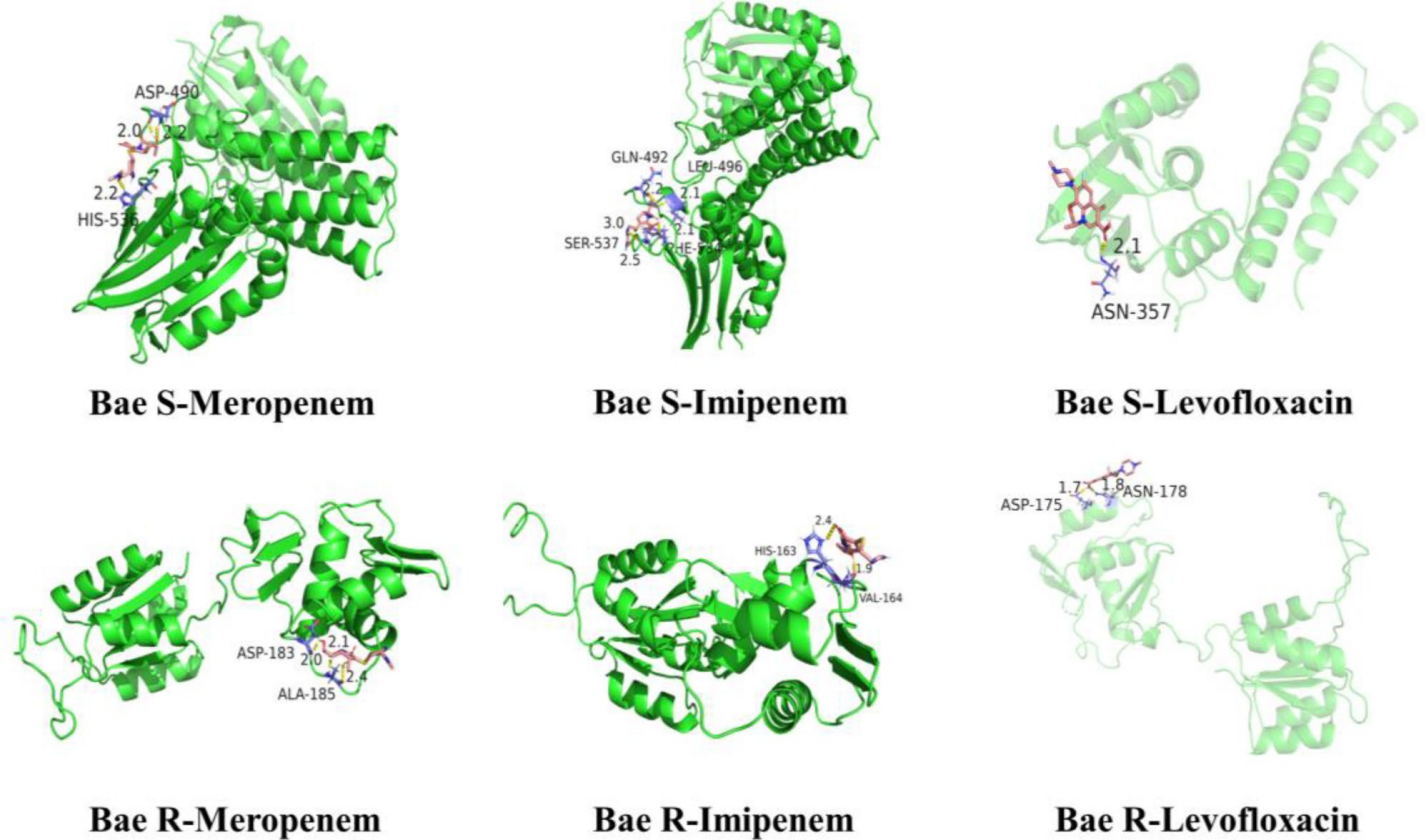
Binding interaction between receptor proteins and three ligands. Green ribbon: receptor proteins; Pink lines: ligands; Yellow dotted line: hydrogen bonds between protein and ligand receptor

Interaction force analysis (Fig. 13): In the Bae S–meropenem docking data, the complex exhibited a hydrogen bond in its active region at HIS-536 with a bond distance of 2.2Å, and two hydrogen bonds at ASP-490 with bond distances of 2.0Å and 2.2Å, respectively. The Bae S–imipenem docking complex revealed single hydrogen bonds at GLN-492, LEU-496, and PHE-534 with bond distances of 2.2Å, 2.1Å, and 2.1Å, respectively, and two hydrogen bonds at SER-537 with bond distances of 2.5Å and 3.0Å, respectively. In the Bae R-meropenem connection data, meropenem established a single hydrogen bond at ASP-183 with a distance of 2.0 Å, and two hydrogen bonds at ALA-185 with bond distances of 2.1Å and 2.4Å, respectively. The Bae R–imipenem docking complex showed two hydrogen bonds in its active region, located at HIS-163 and VAL-164, with bond distances of 2.4Å and 1.9Å, respectively. In the Bae S–levofloxacin docking data, the complex exhibited only one hydrogen bond in its active region ASN-357, with a bond distance of 2.1Å. The Bae R–levofloxacin docking complex revealed a single hydrogen bond for ASN-178 and ASP-175 at bond distances of 1.8Å and 1.7Å, respectively.

### Determination of MIC values and generation of the time-sterilization curve

**Fig. 14:**
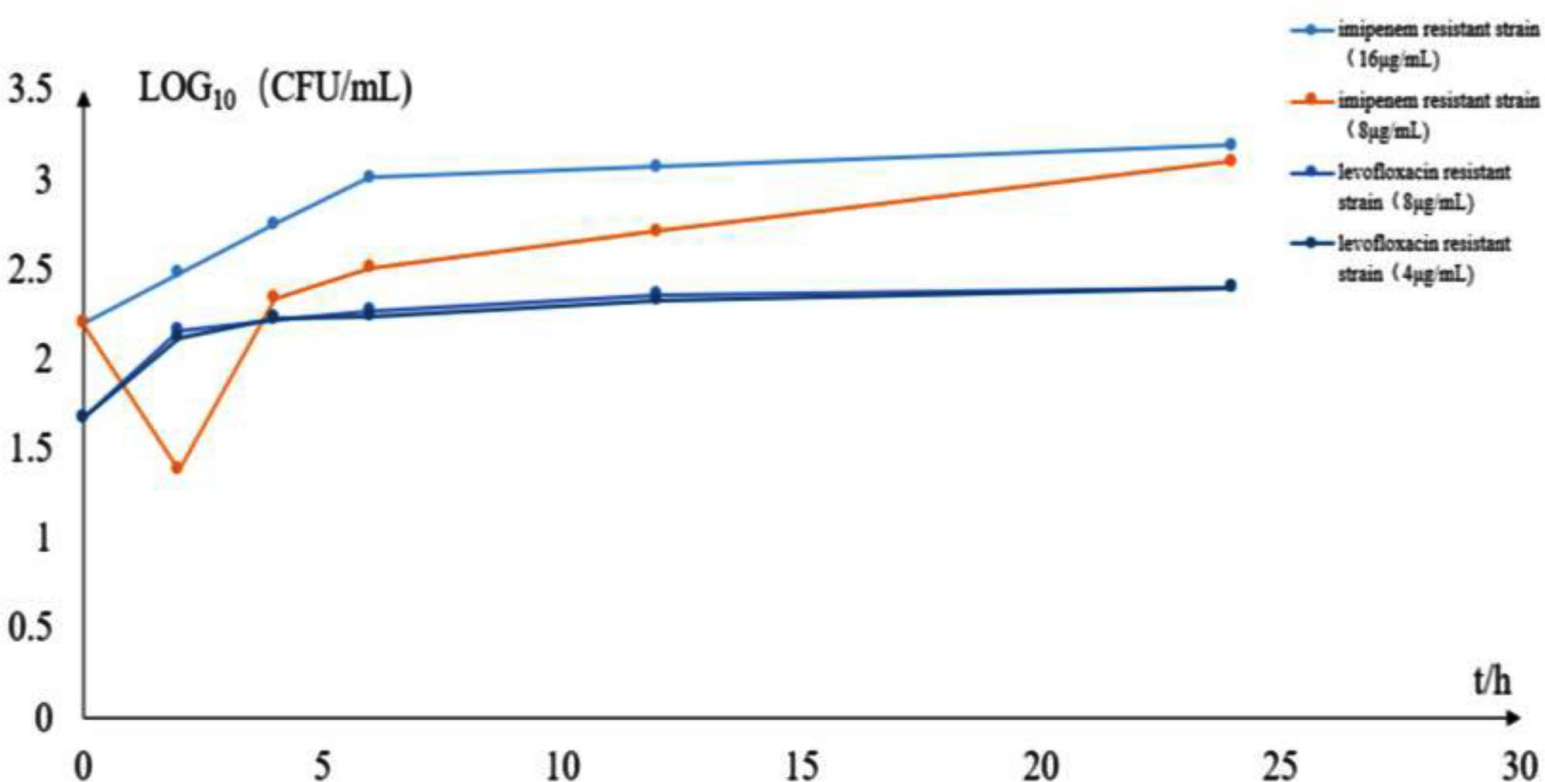
Time sterilization curve of imipenem against CRAB

The MIC values of imipenem for all 10 strains were determined to be 16 µg/mL, whereas the MIC value of levofloxacin was 8 µg/mL. The time-sterilization curves were plotted for specimens treated with ½ MIC of imipenem and ½ MIC of levofloxacin to further compare the differences in drug resistance. The results indicated a significant decrease in the number of colonies at 2 h under the treatment of 8 µg/mL imipenem, demonstrating a certain antibacterial effect. However, this concentration of imipenem could not completely inhibit the growth of specimens, as the number of colonies began to increase after 2 h. However, no bacteriostatic effect was observed at 4 µg/mL levofloxacin (Fig. 14).

### Relative expression of Bae SR in the two-component system

**Fig. 15:**
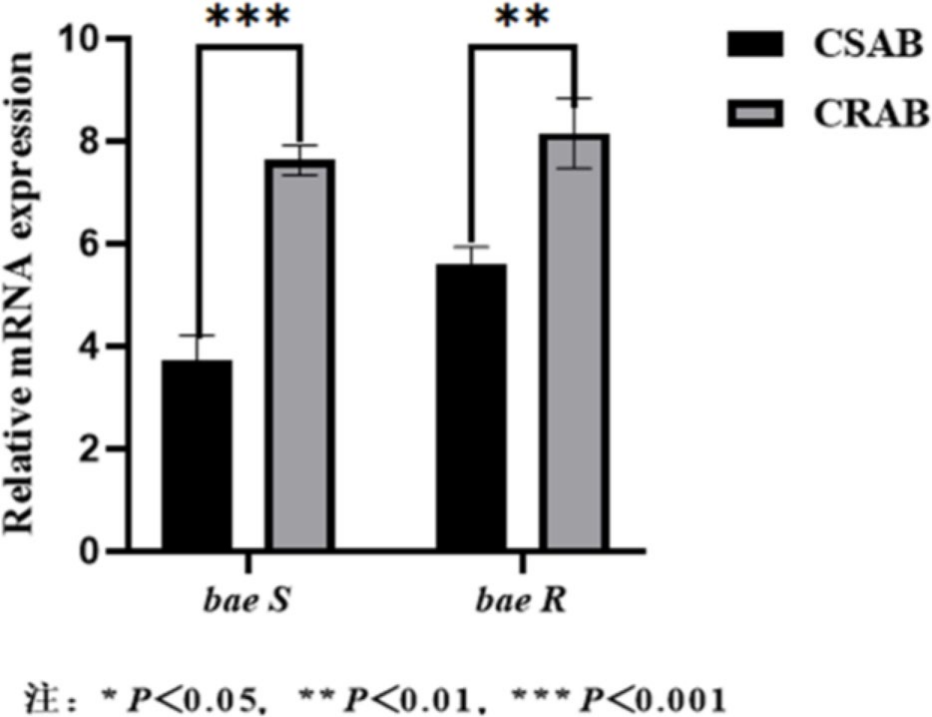
Comparison of *Bae SR* mRNA expression in CSAB and CRAB groups

All 35 *A. baumannii* strains amplified the two-component system genes. *16S rRNA* was used as an internal reference gene to compare the relative mRNA expression levels of the two-component system genes in CSAB and CRAB. The relative expression levels of the *bae S* and *bae R* genes significantly increased in the CRAB group compared with the CSAB group, and the differences were statistically significant (*P*<0.01). A column chart was generated using GraphPad Prism 9 software, and the relative quantitative results are shown in Fig. 15.

## Discussion

The widespread use of various broad-spectrum antibiotics in clinical practice in recent years has led to an imbalance in the body’s normal flora, resulting in an increase in the abundance of clinically drug-resistant bacteria. This trend exacerbated bacterial resistance, resulting in the emergence of multidrug-resistant strains and posing life-threatening risks in severe cases. CRAB is among the most concerning drug-resistant threats currently, highlighting the significance of understanding its resistance mechanisms. Common drug resistance mechanisms [17–20] include the production of carbapenemases, expression of outer membrane efflux pump genes, reduced outer membrane permeability, biofilm formation, and a decrease or lack of expression of outer membrane proteins. Generally, the two-component system responds to antibiotics or indirectly participates in cellular physiological changes, contributing to resistance [21]. TCS typically comprise histidine kinases (HK) and cytoplasmic response regulators (RRs). The reaction mechanism involves receiving a signal stimulus, releasing energy and phosphate groups from ATP, transmitting the signal to membrane-localized sensors with histidine kinase activity, undergoing autophosphorylation, and transferring phosphate groups to corresponding RRs. This process induces conformational changes through histidine residues on HK and aspartic acid residues on RR, leading to specific DNA binding and gene regulation [22]. In this study, the structure and phosphorylation sites of the two-component system Bae SR were predicted. Molecular docking was performed using bioinformatics software with imipenem, meropenem, and levofloxacin. The binding energy of the two-component system Bae SR with carbapenem antibiotics was lower than that with levofloxacin. Also, the hydrogen bonds increased in number and were stronger, indicating better affinity binding. The results of the time-sterilization curve demonstrated a transient bactericidal effect of ½ MIC imipenem. However, with time, the number of colonies increased, possibly due to enhanced drug resistance mediated by the two-component system controlling biofilm formation. In addition, fluorescence quantitative PCR revealed significantly increased expression levels of *bae S* and *bae R* in CSAB and CRAB groups, respectively. The relative expression levels of bae S and bae R significantly increased in the CRAB group compared with the CSAB group. In conclusion, the two-component system Bae SR exhibited remarkable binding affinity with carbapenems, and its overexpression further enhanced carbapenem resistance in *A. baumannii* by promoting biofilm formation. This study provided a theoretical and practical basis for understanding carbapenem resistance in respiratory tract infections caused by *A. baumannii*, offering novel insights for the clinical treatment of *A. baumannii* infections.

## Conclusions

The molecular docking conducted in this study revealed that the combination of Bae SR and carbapenems (imipenem and meropenem) had a strong affinity and greater stability compared with levofloxacin. Additionally, the gene expression analysis of the two-component system in CRAB indicated a significant increase in expression. This suggested that the overexpression of the two-component system genes contributed to enhanced resistance against the carbapenem antibiotics in *A. baumannii*. These findings provided both theoretical and practical insights into carbapenem resistance in respiratory tract infections caused by *A. baumannii*.

## Data Availability

The Bae S and Bae R during the current study are available in the SWISS-MODEL with sequence entries 7CCH (https://www.rcsb.org/structure/7CCH) and 4B09 (https://www.rcsb.org/structure/4B09); While the 3D structure of imipenem, meropenem and levofloxacin were retrieved from the PubChem database, PubChem ID 104838 (https://pubchem.ncbi.nlm.nih.gov/compound/104838), 441130 (https://pubchem.ncbi.nlm.nih.gov/compound/441130) and 149096 (https://pubchem.ncbi.nlm.nih.gov/compound/149096).

## Abbreviations

*Ab*: *Acinetobacter baumannii*
CSAB: Carbapenemase Sensitive *Acinetobacter baumannii*
*CRAB*: Carbapenemase Resistant *Acinetobacter baumannii*
cDNA: Complementary DNA
TCS: Two-component system
HK: Histidine Kinases
RRs: Response Regulators
IPM: Imipenem
MEM: Meropenem
LEV: Levofloxacin
MIC: Minimal Inhibit Concentration
